# A Case of Periodical Neuralgia

**Published:** 1847-01

**Authors:** James L. Taylor

**Affiliations:** Trenton, Ohio


Art. V.—*A Case of Periodical Neuralgia.* By James L. Taylor, of Trenton, Ohio.

Mrs. P., aged 27 years, formerly a resident of a miasmatic district, was attacked, March 2d, 1846, with the most excruciating pains in the left side of her head. Her sufferings were so extremely agonizing as often to cause a temporary loss of reason and consciousness. The first paroxysm, however, was of but short duration. March 3d, at 9 o'clock, the pain returned with rather more than its former violence; and this morning I was summoned to see her. Upon inquiry I ascertained that the week previously she had been indisposed, caused in consequence, as her friends thought, of exposure and loss of sleep. The pain could be traced along the course of the temporo-facial nerve, and was decidedly periodical in its character, and of the quotidian type. She had previously been subject to neuralgia, though of a different nature, the pain having been principally confined to the lower extremities.

After the pain had subsided, transient flushes of heat passed over the face and body; skin intensely hot and dry, attended with thirst, dryness of the mouth, &c., the pulse being full and strong. A good deal of nausea, with occasional vomiting, was also an attendant. In a word, her case presented all the phenomena of a hot stage, such as belongs to intermittent fever, and this was followed by profuse sweating. I directed calomel, grs. xv, and ipecacuan, grs. v, to be given immediately; they acted as an emetico-cathartic. ℞ sulph. quinine grs. xii, sulph. morphia, gr. i—m. ft. div. in pill iv. One to be taken every hour, commencing four hours before the time of the expected paroxysm.

On visiting my patient next morning, I was somewhat surprised at finding that the paroxysm had returned with as much violence as on the day previous. I was informed this morning, that my patient had been, for two years or more, in the habit of using opium, or some of its peparations, freely, for pains of a rheumatic character. As, under these circumstances, it was evident that the opium had, partially at least, lost its influence on her nervous system, I ordered ℞ sulph. quinine, grs. xiv, sulph. morphia, gr. 1¼, to be made into four pills, one to be taken every hour as before directed.

March 5th. The paroxysm again returned, though somewhat abated in violence and duration. In each successive paroxysm, as the pain passed off, the patient experienced a numbness in the part of the head affected, and felt incapable of anything like mental exertion. As the next hot stage came on, the pulse became excited, full, hard and tense, and I determined upon venesection. This was carried to as great an extent as the symptoms rendered expedient, and was followed by calomel, ipecacuan, and opium, in divided doses. Under this plan of treatment the paroxysms ceased to recur, and the health of the patient was soon re-established. She is now in perfect health.

July, 1846.

